# Improving the Treatment and Assessment of Moderate and Severe Pain in a Pediatric Emergency Department

**DOI:** 10.1155/2016/4250109

**Published:** 2016-09-08

**Authors:** Roger Chafe, Debbie Harnum, Robert Porter

**Affiliations:** Division of Pediatrics, Janeway Pediatric Research Unit, Memorial University of Newfoundland, Room 409, Janeway Hostel, 300 Prince Philip Drive, St. John's, NL, Canada A1B 3V6

## Abstract

*Background.* The Janeway Children's Hospital previously enacted a number of measures to improve pain management for patients in its emergency department (ED). While improvements were demonstrated, rates for the timely assessment and treatment of pain remain below standards of care.* Objectives.* The study objectives are to investigate the impact of the previous attempts to improve the treatment of pain and to explore ways to further improve pain management in the ED.* Methods.* Key informant interviews and a focus group were conducted with nurses, physicians, and parents whose children were identified as having severe pain.* Results.* Interviews were conducted with 31 parents or children, 9 physicians, and 8 nurses. The focus group was attended by 15 nurses. Previous initiatives were viewed as improvements. Continued barriers include difficulties in accurately capturing the level of pain, issues in treating pain for specific types of patients, and inadequacy in addressing patients in severe pain.* Conclusion.* Changes in pain treatment protocols can result in positive impacts but are likely insufficient on their own to achieve desired standards of care. Consistent measurement and engagement with staff can identify additional opportunities for improving pain management within an ED setting.

## 1. Introduction

The assessment and treatment of pain are important aspects of pediatric emergency care, with inadequate pain management having the potential for lasting negative outcomes for patients [1, 2]. Formal pain assessment within a pediatric emergency department (ED) is the standard of care, with multiple self-reported and behavioural scales existing for children of different developmental capacities [3]. However, pain scoring has not consistently produced positive results in terms of better pain treatment [4–6], with low correlation between pain intensity assessment at triage and the provision of an analgesic [7]. As in other healthcare settings, proven treatments for pain in the pediatric ED are also often underutilized [8, 9].

The Janeway Children's Health and Rehabilitation Centre is the only tertiary care children's hospital in the Canadian province of Newfoundland and Labrador (NL). Its ED has approximately 34,000 patient visits annually, representing a wide range of pediatric conditions and injuries. In 2011, we examined the timely treatment of pain for patients with supracondylar humerus fractures in the ED, finding that in only 15% of cases did patients receive an analgesic within 60 minutes of triage [10]. In response, in 2012, the ED instituted a triage pain assessment and treatment medical directive for mild to moderate pain [11]. The medical directive allows nurses, including those involved in the initial triaging of patients, to treat mild to moderate pain with ibuprofen or acetaminophen without requiring preapproval or prior assessment of the patient by a physician. Along with the directive, the ED instituted a policy which mandated the documentation of self-reported pain scores for all children presenting with pain to the ED. These changes were supported by educational interventions aimed at nurses, residents, and physicians working in the ED to improve their awareness of treating pain, including training sessions, posters, and other reminders to prompt staff to treat pain in a timely manner.

As part of an internal evaluation of these multiple initiatives, we compared the analgesic treatment of children with acute supracondylar fractures after these interventions with our previous study data. We found that the proportion of patients with acute supracondylar fracture treated with an analgesic within 60 minutes of triage increased since the medical directive became enacted from 15% to 54% [12]. While a definite improvement, this result showed that still almost half of these patients with a condition typically associated with moderate to severe pain were left untreated after 60 minutes. Furthermore, a small evaluative chart review conducted within the ED of a representative sample of visits from August 22, 2013, to February 3, 2014, showed that pain scores were still rarely documented, even though the expectation was that pain scoring (either identified as mild/moderate/severe or on a scale from 1 to 10) was to be done on all patients presenting with any pain.

In this article, we explore the current barriers to improving the assessment and treatment of pain within our pediatric ED. The study focuses on three main issues: (1) how well the previous attempts to improve the treatment of pain in the ED are working in practice; (2) current barriers to further improving of the assessment and treatment of pain in the ED; and (3) the feasibility of expanding the current medical directive to include the use of opioids, most likely intranasal fentanyl, to allow nurses to treat patients with more severe pain. Fentanyl is an opioid analgesic which, when delivered intranasally, has a very quick onset of action (less than 10 minutes) and a duration of action of less than an hour [13]. Its use by nurses could allow for quick relief of pain while further care, including other analgesic measures, may be undertaken. This study is a real world example of the multiple issues faced by the different stakeholders within a pediatric ED when trying to improve the treatment of pain for its patients. It highlights both the accomplishments and the challenges of improving pain management within a dynamics clinical environment. It hopefully provides useful insights for enacting similar initiatives in other pediatric EDs.

## 2. Methods

### 2.1. Design

The project followed a qualitative, evaluative approach [14]. Data was collected through one-on-one key informant interviews and one focus group held with nurses involved in triaging patients in the ED. Physician and nurse interviews and focus groups were conducted using a semistructured interview guide (see Appendix 1 in Supplementary Material available online at http://dx.doi.org/10.1155/2016/4250109), which was developed by the research team to reflect the study objectives. Interviews with parents followed a similarly developed structured interview guide (see Appendix 1). Interviews and the focus group were all conducted in person by one member of the research team (DH). Ethics approval for the project was obtained from the* Newfoundland and Labrador Health Research Ethics Authority* [15].

### 2.2. Participants

This study involved engaging three key groups related to the treatment of children with pain in a pediatric ED: ED nurses, ED physicians, and parents whose children were identified as having severe pain in the ED. For parent interviews, triage nurses notified the research nurse on days she was available when a patient with a pain scale of 8–10/10 or “severe” was triaged. We focused on children in severe pain because they were not covered by the current medical directive and we wanted to gauge their support of using opioids as an analgesic, as these medications would likely be part of any expansion of the current medical directive to better serve these patients. The research nurse was available for 19 eight-hour shifts during the study period to meet with patients, with these days being randomly selected over the study period, including on weekends. Once identified, the parent was approached by the research nurse for consent to be interviewed. All nurses and physicians who were working regularly within the ED at the time of the study were invited to participate in the study. For interviews with physicians, recruitment was conducted by departmental e-mail. For interviews with nurses, recruitment was conducted by a poster in the ED and direct approach of individual nurses by a study researcher. The focus group was held as part of a nurse education day for ED nurses and included all those who were interested and consented. All interviews and the focus group were conducted between March 27 to June 20, 2015.

### 2.3. Data Collection and Analysis

All physician and nurse interviews and the focus group were recorded and professionally transcribed. Because parent interviews were conducted within the ED using a structured guide and were meant mostly to gauge their satisfaction with their child's pain treatment, only field notes, which did include direct quotes, were taken of the responses.

Analysis was conducted using an applied thematic analysis approach [16]. For the structured parent interview questions, participant responses to each question were collected together and analyzed by question. For the semistructured questions, transcripts were coded using NVivo 10 software [17]. All codes and themes were developed inductively through analysis of the data [18]. Key themes were developed and clarified through the discussions with the research team. No names or identifiers were used in reporting results to ensure anonymity.

## 3. Results

We conducted 28 interviews with parents or legal guardians of children identified as presenting in severe pain to gauge their current level of satisfaction with pain management in the ED. Three additional interviews were conducted with teenage patients. In two cases, the parent indicated that the teenager (both aged 17) would be the best person to answer the interview questions. The teenagers then answered the interview questions in the presence of the parent. In another case, the patient was unaccompanied and self-identified as an emancipated minor and was interviewed alone. We thus had 31 interviews with either parents or the youth patients. To help ensure anonymity, we analyzed all of these interviews together. To avoid confusion, we refer to them as parent interviews subsequently in the article even though they include three interviews which were directly with patients, as they were all initially intended to be interviews with parents or guardians. There were 16 physicians and 27 nurses working in the ED at the time of the study. We conducted 17 key informant interviews, 9 with physicians and 8 with nurses who worked in the ED. We conducted one focus group with triage nurses, which was attended by 15 nurses, including one nurse who was also interviewed. This represents that 81.5% of the nursing staff and 56.3% of the physicians working at the ED participated in the study.

### 3.1. Parent Interviews

The children of the parents interviewed in the ED ranged in age from 3.3 to 17 years of age, with an average age of 12.5 years. The conditions represented included 15 (48.4%) types of trauma cases, including 6 (19.4%) fractures or dislocations cases, 4 (12.9%) other musculoskeletal trauma cases, 2 (6.5%) head injury cases, and 3 (9.7%) other or multiple trauma cases. Nontrauma cases included 9 (29.0%) abdominal or flank pain cases, 3 (9.7%) nontraumatic back pain cases, and 4 (12.9%) other nontrauma cases. 30 of 31 (96.8%) parents felt that their child's self-reported score accurately reflected their child's level of pain. Nine (29.0%) parents reported their child received some type of analgesic before presenting to the ED. In the ED, 9 (29.0%) patients received ibuprofen, 7 (22.6%) fentanyl [6 intranasal (IN), 1 intravenous (IV)], 2 (6.5%) acetaminophen, 2 (6.5%) ketorolac, 2 (6.5%) subcutaneous morphine, 2 (6.5%) combinations of medications, and 7 (22.6%) no medication for pain in the ED.

Parents were asked how satisfied they were with the management of their child's pain. This was an open ended question, to which 19 parents said that they were “very satisfied” and 12 said that they were “satisfied” with the pain management that their child received. As one parent said about her child and herself, “we were really satisfied with the pain treatment at Janeway today. Everybody worked quickly to help with medication and the backslab was done quickly.” One patient commented on the improvement in the pain management at the ED. “I was seen here with a broken arm a couple of years ago and received nothing for pain. I am very satisfied with today's treatment.” One issue for people who only expressed being satisfied was with the effectiveness of the medication they received. As one patient who received morphine said, “I was very satisfied, treated quickly, but would have liked to get a better result from the pain treatments.” Another issue expressed by two parents was that their child was given ibuprofen first, which did not address the pain. They were then given a second medication which reduced the pain, and they “would have preferred something stronger at the beginning of their visit.” Another patient who only expressed being satisfied said her pain medication was being delayed until after her underlying condition is diagnosed. “I am satisfied with the treatment. I haven't had anything for pain here as I took some Aleve before coming out. It has dulled the pain a little now. They are trying to figure out if I have appendicitis. So I don't think they want to give me anything else.”

Parents were asked if they would be comfortable with their child being given an opioid to treat their severe pain if recommended to them by ED staff. 30 of 31 (96.8%) parents said that they would allow their child to receive an opioid for severe pain. The opinion shared by many parents was that “I would not want my child to be in pain, particularly severe pain.” It should be noted that for 13 of 31 (41.9%) parents interviewed, their child did receive some form of opioid during their treatment. The only parent who said that they would not want their child to receive an opioid cited concerns about addictions as the main reason for being opposed: “No, unless the pain was unbearable and I would have to know which opioid, as I worry about addiction to opioids.” While saying they were supportive of opioids, other parents also expressed some concerns about possible addiction and felt that opioids should only be used for very severe pain.

### 3.2. Nurse and Physician Assessment of Performance

In terms of staff perceptions around how well the ED was currently performing in assessing and treating pain, views ranged from “moderately well” to “fair to poor.” One participant distinguished between the assessment and management of pain. “I think that the assessment of the pain is probably good – I think it's maybe the management of the pain… that might be more the issue.” Several respondents, without being given a scale beforehand, felt that an overall score of “7 out of 10” was a reasonable assessment of the ED's current performance. No negative effects relating to the implementation of the medical directive to treat mild to moderate pain or the previous education initiatives were reported in the interviews or focus group. While the initial attempts to improve pain management appear to be supported, many respondents felt that improvements could be made so that pain is “appropriately managed to the best of its [ED's] ability.”

### 3.3. Factors Impacting Pain Management

ED nurses and physicians identified a number of issues which they felt negatively affected pain management in the ED; see the following list:

- *Lack of awareness that performance is still a problem.*
- *Staffing/patient flow issues.*
- *Being too focused on medication.*
- *Type of medical condition impacting pain treatment.*
- *Accuracy of the pain assessment.*
- *Current medical directive which does not address severe pain.*

The first issue was the belief by many participants, particularly expressed in the focus group, that the previous initiatives were more successful in addressing the issues of pain management and a lack of recognition that the department was still underperforming in assessing, recording, and appropriately treating pain. As one focus group participant said, “my perception was that more people were implementing it,” in terms of both recording a pain score and giving medication during triage. Other participants likewise said that they thought that the rates of following the adoption of the new protocol were higher. Others were under the impression that “everyone has been implementing it, right?”

There was the perception, particularly amongst some of the nurse participants, that organizational issues and patient flow negatively impacted the department's performance. Factors such as the number of patients presenting at a particular time were identified as impacting timely pain assessment and treatment; “If it's busy, we are definitely doing a poor job. If it's not very busy and they can be seen quickly, then we probably do a fair job.” Similarly, staffing levels were identified as impacting on performance.

Two physicians said that the previous pain initiatives “focus on medications” in pain treatment, excluding other methods of pain reduction, for example, using a back slab or splinting injuries, when in many cases “splinting will take care of 90% of the pain.” They also felt that future initiatives, for example, training sessions, should be more inclusive of nonpharmacological methods to reduce pain.

Many participants felt that the type of injury played a role in whether timely treatment was administered: “I wonder if they [acute abdomens] are being assessed appropriately, and even simple things, like ear pain, when it presents.” A number of factors were identified as impacting on pain treatment for conditions like abdominal pain. One participant felt that in some situations “nurses feel uncomfortable treating the condition,” for example, abdominal pain and fractures, without physician involvement. In other cases, “nursing staff don't have the ability to treat that pain in triage,” partly due to restrictions on what pain medications they can prescribe. With abdomen pain, there can be issues around whether the patient should get oral, IV, or intramuscular (IM) medication, which may require physician consultation. In other cases, there was a feeling that “consultants don't want belly pain treated before they see” the patient, fearing that the medication may hamper diagnosis. Finally, situations in which patients have potential or queried fractures but a definite diagnosis has not been made are another “area that is grossly overlooked.”

Another set of concerns raised by staff related to relying on pain scores reported by the patient or their parent. Many staff questioned the value of relying solely on self-reported scores, particularly with children and youth who may not have a range of experiences with pain to compare. As one nurse participant said, “If we're gonna use a scale, I think we need to find a scale that is gonna be valid with our population. For example, like in older kids, just tell them to go ‘1 to 10' is really not a good measure of pain. We'll have some kids come in and they're very stoic and they'll be like, 6 or 7 and… and they've got appendicitis and I've got other kids who are sitting up texting saying, ‘oh yea, my pain is 8 outta 10.' So there would have to be something, a scale used other than the patient's report of pain.” There was some dispute around how scores are reported, with at least one participant saying in the focus group that they adjust the assigned score to reflect not only the person's self-reported score, but also the nurse's assessment of behaviour and clinical condition. Other participants said that they recorded the observations about the likely level of pain associated with a condition and the behaviour in their clinical notes. As one of the nurses said “what they need to change, is bring in a scale that covers [the] clinical condition, what the parents are saying, what, you know, bring it all into the picture.”

While there was a good deal of support for the provision of the medical directive, many participants felt that it did not go far enough. Suggested improvements include ensuring that “the triage nurse had a broader range of medications they could give to help treat pain,” including expanding the range of medications that could be administered under the current department protocol. In terms of extending the current medical directive, all physician respondents said that they would be comfortable with expanding the medical directive to include allowing nurses to administer intranasal fentanyl if there was a clear protocol for doing so and if nurses had received appropriate training. One participant did qualify that their level of comfort would depend on the specific nurse, for example, their level of experience and length of time working in a pediatric ED. This protocol and training would need to address issues related to other medications, allergies, and some restrictions on nurses providing fentanyl for certain medical conditions. While there was no consensus on what conditions for which there should be restrictions, patients with head injuries, headaches, altered level of consciousness, or cognitive delays were most commonly mentioned. Other conditions that participants had some concerns about allowing nurses to treat them independently included respiratory distress, younger patients, patients who took any medications at home for pain, multisystem trauma, and abdominal pain. Beyond those situations, participants felt that “the vast majority of patients that we'd have come through could have the pain treated effectively with the nurse.” Training on how to discuss opioids and educate parents prior to giving an opioid was also seen as useful.

## 4. Discussion

In this article, we present a real world example of the use of different mechanisms to improve the assessment and the treatment of pain within a pediatric ED. The study benefited from being part of a series of studies addressing the issues of pain management within the same ED and the high level of participation from both the nursing and physician staff. We found that there was already a high level of satisfaction with the parents interviewed with the care their child received. Both nursing and physician staff generally saw initiatives that have already been enacted as effective, including a medical directive allowing nurses to treat mild or moderate pain without preapproval by a physician. For pediatric centres which do not allow for nurses to treat mild to moderate pain, our experience suggests that this is an effective method for improving the timeliness of treating within the pediatric emergency setting with no negative issues being reported since this medical directive has been adopted.

One issue raised by staff was that the current medical directive does not allow nurses to treat patients with severe pain, which are often the patients in the most need of immediate relief. This issue is not unique to our ED, as the focus for triage-administered analgesics in pediatric care has been mostly on patients with mild to moderate pain [19, 20]. Although they were not asked directly about it being administered by a nurse, the vast majority (96.8%) of parents of children with severe pain interviewed said that they would be supportive of their child having an opioid given to their child if they were in severe pain. Given that this intervention is meant to improve the treatment for patients with more severe pain, it is interesting that these patients also report fairly high levels of satisfaction with current pain management. One issue of concern raised by parents was the fact that milder pain medications were given in some situations, rather than going straight to a stronger pain medication. For children with severe pain, initial analgesic therapy should generally involve an opioid and extending the current medical directive could improve the timely administration of these medications.

Parents felt that the self-reported pain score given by their child accurately reflected their child's level of pain. This perspective contrasts with some of the comments made by staff about the need to adopt a more holistic approach to the assessment and documentation of pain. One aspect which may account for this difference is that parents interviewed in this project were solely those who were experiencing severe pain, which may be more clearly evident. Parents may have played a role in assessing or approving the pain score that was reported, particularly for very young children. Interviews were also conducted with the children present, which may also have made parent hesitant to contradict their children's assessment.

There are potential ways to address some of the concerns raised by staff. Considerable discomfort was expressed with treatment of pain arising from certain specific clinical conditions, for example, headache, abdominal pain, and multiple trauma. One approach to this problem would be to develop protocols specific to these conditions, as well as others where pain treatment does not typically follow the usual pathway (e.g., migraine in a known migraineur might be handled differently). It is important that subsets of patients are not unduly disadvantaged simply because of their medical condition. One important group of patients are those presenting with severe pain. Both healthcare providers and parents/patients appeared to support early treatment with intranasal fentanyl for identified severe pain; healthcare providers were comfortable with a nurse-administered dose of this medication as long as appropriate protocol was in place. The issue of patients' experience of different routes of administering analgesics has not been well studied. While some patients may find intranasal administration of medications distressing, this route likely compares favorably to other routes appropriate for acute severe pain, such as intramuscular injection [21]. For a child who does not otherwise require intravenous access, intranasal administration has the advantages of avoiding the pain of an IV start as well as reliable prompt administration, avoiding the delays often associated with intravenous access. Intranasal fentanyl should be strongly considered as an option for these patients. Other initiatives that could be tried include including training on nonpharmaceutical interventions in initiatives to improve pain management, identifying ways of standardizing the recording of pain scores to allow for patient self-reports and clinical and behavioural assessments made by staff, and making extra efforts to remind staff of the importance of proper pain management during periods of operational stress, for example, during staff shortages or high patient volumes.

While the article gives a real world example of the issues which a pediatric emergency faces in improving its pain treatment, the study is limited to a single centre, and concerns of stakeholders at other sites may vary based on different cultural or other factors. Scheduling issues meant that the research nurse was only able to do interview with parents on selected days during the study period. We thus do not claim that these interviews are a representative sample but do give a picture of the level of satisfaction of a group of patients. As discussed above, the parent interviews included three interviews which were conducted with teenaged children, rather than parents. Patient interviews were with parents of children with severe pain and may not reflect the views of patients with milder forms of pain. Issues may also be different between an academic pediatrics ED and other centres treating emergency pediatric cases.

## 5. Summary

This study provides useful information on some of the barriers to effective delivering of appropriate and timely analgesic interventions to children presenting to EDs. While initial initiatives showed improvements in timely pain management, given the current discrepancies between adherence to pain policy and perception by caregivers, ongoing feedback and engagement as part of a continued quality assurance program would be useful. There appear to be ongoing concerns regarding the accuracy of a self-report amongst caregivers, although this was not shared by the small sample of parents in the study. While the concerns of healthcare providers should be acknowledged, providers must be encouraged to give due consideration to a self-report of pain score while exercising clinical judgement. Potential ways to address some of the concerns raised by staff include protocols specific to particular conditions, developing an expanded medical directive to allow staff to treat patients with severe pain, including nonpharmaceutical interventions in training initiatives to improve pain management, and better addressing pain management issues during periods of operational stress, for example, during staff shortages or high patient volumes.

## Supplementary Material

The supplementary material includes the interview guides used with health providers (both nurses and physicians who worked in the ED), and with the parents of patients who presented to the ED in severe pain.
