# Supplementary material for: Improving the Treatment and Assessment of Moderate and Severe Pain in a Pediatric Emergency Department
Source: Pain Res Manag. 2016 Sep 8;2016:4250109. doi: 10.1155/2016/4250109 (PMC5031872; doi:10.1155/2016/4250109)
Supplement: Supplementary file 1 — The supplementary material includes the interview guides used with health providers (both nurses and physicians who worked in the ED), and with the parents of patients who presented to the ED in severe pain. [file 4250109.f1.docx]

Appendix 1: Interview and Focus Group Questions

**Physician and Nurse Interview and Focus Group Questions**

How well do you think that pain is currently being assessed in the Janeway?

How could this be improved?

Are there groups of patients or clinical conditions where pain is not well assessed?

Are you comfortable with nurses assessing pain and treating severe pain with intranasal fentanyl according to a protocol? If not, why not?

Are there any specific groups of patients that you would be concerned about with such a protocol?

Are there any clinical conditions where you would be concerned about such a protocol being instituted? In yes, elaborate on each of the above.

What level of intensity of pain (1-10), assuming it is accurate, so you think should be treated with a narcotic (intranasal fentanyl) as opposed to a simple analgesic (ibuprofen or acetaminophen)?

Do you have any experiences/perceptions with/regarding other IN medications?

What influence does the IN route have on timing?

Do you have concerns about side effects or monitoring?

**Parent Interview Questions**

Would you describe your/your child’s pain as mild, moderate or severe?

Your/your child’s pain was rated as x/10. Do you think this accurately reflects your/your child’s pain?

Your/your child’s pain was treated with XXX. Are you satisfied with this treatment?

Overall, how satisfied were you with your/your child’s pain treatment?

If pain is severe, Tylenol or Advil is sometimes not strong enough and a narcotic is used. This is a safe and effective form of treatment. Would you be comfortable for you/your child to be treated with such a medication if his/her pain was severe?
